# Defining the Enablers and Barriers to the Implementation of Large-scale, Health Care–Related Mobile Technology: Qualitative Case Study in a Tertiary Hospital Setting

**DOI:** 10.2196/31497

**Published:** 2022-02-08

**Authors:** Ravi Aggarwal, Sheena Visram, Guy Martin, Viknesh Sounderajah, Sanjay Gautama, Kevin Jarrold, Robert Klaber, Shona Maxwell, John Neal, Jack Pegg, Julian Redhead, Dominic King, Hutan Ashrafian, Ara Darzi

**Affiliations:** 1 Institute of Global Health Innovation Imperial College London London United Kingdom; 2 Department of Surgery & Cancer Imperial College London London United Kingdom; 3 Department of Computer Science University College London London United Kingdom; 4 Imperial College Healthcare NHS Trust London United Kingdom

**Keywords:** mHealth, implementation science, mobile technology, mobile apps, clinical applications, smartphone apps, health care industry, stakeholders, mobile phone

## Abstract

**Background:**

The successful implementation of clinical smartphone apps in hospital settings requires close collaboration with industry partners. A large-scale, hospital-wide implementation of a clinical mobile app for health care professionals developed in partnership with Google Health and academic partners was deployed on a bring-your-own-device basis using mobile device management at our UK academic hospital. As this was the first large-scale implementation of this type of innovation in the UK health system, important insights and lessons learned from the deployment may be useful to other organizations considering implementing similar technology in partnership with commercial companies.

**Objective:**

The aims of this study are to define the key enablers and barriers and to propose a *road map* for the implementation of a hospital-wide clinical mobile app developed in collaboration with an industry partner as a data processor and an academic partner for independent evaluation.

**Methods:**

Semistructured interviews were conducted with high-level stakeholders from industry, academia, and health care providers who had instrumental roles in the implementation of the app at our hospital. The interviews explored the participants’ views on the enablers and barriers to the implementation process. The interviews were analyzed using a broadly deductive approach to thematic analysis.

**Results:**

In total, 14 participants were interviewed. Key enablers identified were the establishment of a steering committee with high-level clinical involvement, well-defined roles and responsibilities between partners, effective communication strategies with end users, safe information governance precautions, and increased patient engagement and transparency. Barriers identified were the lack of dedicated resources for mobile change at our hospital, risk aversion, unclear strategy and regulation, and the implications of bring-your-own-device and mobile device management policies. The key lessons learned from the deployment process were highlighted, and a road map for the implementation of large-scale clinical mobile apps in hospital settings was proposed.

**Conclusions:**

Despite partnering with one of the world’s biggest technology companies, the cultural and technological change required for mobile working and implementation in health care was found to be a significant challenge. With an increasing requirement for health care organizations to partner with industry for advanced mobile technologies, the lessons learned from our implementation can influence how other health care organizations undertake a similar mobile change and improve the chances of successful widespread mobile transformation.

## Introduction

### Background

The implementation of mobile technologies in hospital workflows has the potential to significantly improve patient safety, transform health care delivery, and positively affect patient outcomes [1]. Although there is widespread agreement about the importance and potential benefits of mobile technologies to tackle critical challenges in health care, successful implementation of this emerging technology in clinical settings has proven to be challenging [2-4]. In the National Health Service (NHS), the deployment of hospital-wide clinical mobile apps in secondary care is still uncommon despite the ubiquity of smartphone ownership among health care professionals (HCPs) [5,6]. HCPs continue to use their own smartphones in hospitals for daily clinical tasks, including communication among teams, accessing clinical apps such as decision support aids or medical calculators, and educational purposes [7-10]. Although medical apps are freely available for download from *App Stores*, there has been limited deployment of hospital-wide clinical mobile apps for HCPs in the NHS.

Streams (Google Health) is a multifunctional smartphone app displaying a range of patient clinical information that was implemented on a *bring-your-own-device* (BYOD) basis at our hospital (Imperial College Healthcare NHS Trust [ICHNT]). Streams was iteratively developed by a multidisciplinary team of researchers, clinicians, and developers in a tripartite partnership with DeepMind Health and Google Health [11] and Imperial College London (ICL). This partnership was initiated as part of ICHNT’s goals to be one of the most digitally mature organizations in the NHS and one of 16 Global Digital Exemplar providers in the United Kingdom.

The ambition was to process and display routinely collected clinical results on the clinicians’ own smartphone devices through integration with the hospital’s existing information systems and electronic health records (EHRs; Cerner Corporation). Streams was developed for use on iOS devices only as it has been demonstrated that 75.6% of physicians and 58.4% of nurses at our institution use an iOS device [5]. Streams was registered with the Medicines and Healthcare Products Regulatory Agency as a Class I, nonmeasuring, nonsterile medical device under the EU Medical Device Directive (1993). The implementation of the Streams app at our hospital began in early 2019 with a small pilot group of clinicians. Further development of the app and instigation of mobile device management (MDM) software ensued before widespread deployment commenced in January 2020 with the app available to all HCPs across each hospital site within the organization.

Before Streams was implemented at our hospital, a limited version of the app had been deployed in a focused capacity at another London-based hospital network to aid HCPs in the detection and management of acute kidney injury [12]. This deployment attracted significant public and media interest because of an investigation by the Information Commissioner’s Office (ICO) into the nature of data processing between the Trust and the industry partner [13-15]. In a separate initiative that learned from these well-publicized issues, a wide-scale implementation of the Streams app was undertaken at our hospital. To the best of our knowledge, this was the first large-scale, hospital-wide deployment of a BYOD clinical smartphone app using MDM technology in the NHS. As such, the implementation process provided important insights into the opportunities and challenges of delivering this type of innovation to the NHS and health systems more widely.

### Objective

In light of previous experiences, the difficulties encountered and the *lessons learned* from this deployment may be generalizable and applicable to other health care organizations that are considering working with industry partners as data processors to deploy similar mobile technology and with academic partners to independently evaluate these interventions. Therefore, the aim of this study is to characterize the key enablers and barriers and to propose a *road map* for the implementation of a hospital-wide clinical mobile app developed in collaboration with an industry partner as a data processor and an academic partner for independent evaluation.

## Methods

### Design

Semistructured interviews were conducted individually with the study participants at a single time point. An in-depth literature review was undertaken to identify implementation and change management frameworks applicable to digital health interventions. These findings led to the creation of a structured topic guide that drew heavily from the *Digital Change in Health and Social Care* document published by The King’s Fund in 2018 [16]. This seminal report highlights five key areas to consider when undertaking digital change implementation in health care: leadership and management, user engagement, information governance, partnerships, and resourcing and skills. These areas formed the domains upon which the enablers and barriers were characterized.

### Participants and Sample Size

The participants were purposively recruited [17] following a key informant strategy [18] to ensure that a well-informed, representative sample of staff members was obtained from all participating stakeholders who were involved in the implementation of the Streams app. These were predominantly members of the Streams Steering Group (SSG), which consists of academics, clinicians, and technicians from ICHNT, the Institute of Global Health Innovation at ICL, and Google Health.

The total number of individuals who were involved in the implementation process was small, which unavoidably restricted the number of interviewees. The sample size was guided by repeated assessments of the emerging data and in line with international consensus guidance and previously published work [19,20]. Although the sample was necessarily heterogeneous to ensure sampling of all the various roles in the steering committee, the wider research team agreed that the data set was adequate for the stated objectives to be met [21]. In total, 16 members of the steering committee were invited to participate in the study by email, with 14 (88%) consenting to be interviewed. All 3 participating organizations were adequately represented. Each participant was interviewed once. The mean duration of each interview was 35.53 (SD 12.36) minutes, and a total of 497 minutes of audio recordings were transcribed for analysis.

### Data Collection and Analysis

All interviews were conducted on the web with the participants over Microsoft Teams videoconferencing software. Audio recordings were made of the interviews, which were then transcribed verbatim. All interviews were conducted by a single male researcher (RA) who is a practicing physician and conducted the study as part of a wider research project. The interviewer took field notes during the interview, which were used to adapt the interview guide depending on the verbal responses given.

A broadly deductive approach to data analysis was used [22], with the topic guide adapted, as noted, from the King’s Fund *Digital Change in Health and Social Care* [16] document that formed the basis of an initial predefined coding framework and, thus, a consistent focus for interpretation. The analysis was conducted by 2 independent researchers (RA and SV). After familiarization with the data, an iterative process of coding and indexing was adopted to ensure that important aspects of the data were not missed from the predefined coding framework. A working analytical framework was developed and applied to the coding of all the transcripts. The coded data were then charted to emerging themes, which were then summarized into the framework matrix. All data were coded, indexed, and charted using NVivo for Mac v12 (QSR International).

### Ethical Considerations

Ethical approval was granted for this study by the Joint Research Compliance Office at ICL under the Science Engineering Technology Research Ethics Committee process (reference 20IC5854). Informed consent was obtained from all the participants. All data were deidentified for the purposes of analysis, with each individual interview identified by an alphanumeric code. The participants were acknowledged only by their organization to avoid the identification of specific participants.

## Results

### Overview

**Figure 1:**
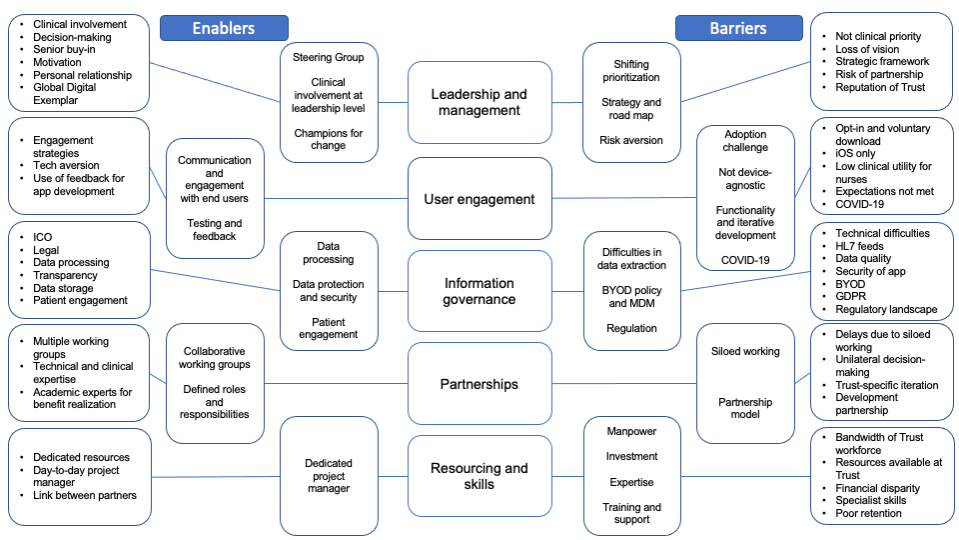
Thematic map for semistructured interviews demonstrating developed subthemes and overarching core themes. BYOD: bring-your-own-device; GDPR: General Data Protection Regulations; HL7: Health Level 7; ICO: Information Commissioner’s Office; MDM: mobile device management.

The reported enablers and barriers to the implementation of Streams at ICHNT are described across the 5 key themes in successful digital change management [16]. The development of overarching themes and subthemes is summarized in Table 1 and Figure 1*.*

**Table 1:** Reported enablers and barriers to the implementation of the Streams app.

Theme	Enablers	Barriers
Leadership and management	Steering Group Clinical involvement at leadership level Motivation and champions for change	Shifting prioritization Unclear strategy and road map Risk aversion
User engagement	Communication and engagement with end users Testing and feedback	Adoption challenge Not device-agnostic Functionality and iterative development COVID-19
Information governance	Data processing and information sharing Data protection and security Patient engagement and transparency	Difficulties in data extraction BYOD^a^ policy and MDM^b^ Regulation
Partnerships	Collaborative working groups Defined roles and responsibilities	Siloed working Partnership model
Resourcing and skills	Dedicated project manager	Personnel Investment Expertise Training and support

^a^BYOD: bring-your-own-device.

^b^MDM: mobile device management.

### Leadership and Management

The involvement of senior leadership in the implementation process helped with decision-making, highlighted the importance of the project within the organization, and helped motivate other key stakeholders in the process. Among the enablers identified was the establishment of the SSG, consisting of key stakeholders and leaders from all 3 organizations, including the Chief Clinical Information Officer (CCIO), Caldicott Guardian, and Medical Director from the Trust; UK Lead and Project Managers from Google Health; and the Chief Scientific Advisor at the university partner. The SSG met every 6 weeks with an overarching remit to manage the project with decision-making by consensus. Clinical leaders were also recruited to the SSG and were able to act as champions for change among clinical teams:

I think the steering committee overall worked well. It had sort of senior people from both parties that met on a regular basis. I think it was important that there was that senior buy in [...] the senior people from both parties regularly engaged, despite busy schedules.

Participant #13, Google Health

There's a lot of clinical involvement and I think that ends with a product that is, at its core, clinically safe and has clinical utility.

Participant #7, Google Health

Barriers identified at a leadership level included shifting prioritization of the project, competing with a myriad of other information technology (IT) projects ongoing at the hospital, which affected the amount of dedicated resources allocated:

I think because Streams was not a key clinical system [...] you would not expect streams to be prioritized over other key clinical systems, obviously.

Participant #13, Google Health

An unclear strategic framework for deployment, risk aversion, and extensive due diligence caused by the alliance with a high-profile industry partner were also identified as barriers:

What there wasn't was almost that strategic framework within which to sit it, and I think we were both to blame for that, and actually possibly had either side pushed the other one a bit harder on that, that would have helped, but I think we were both a bit amateur on that front.

Participant #1, ICHNT

### User Engagement

Multiple strategies were trialed in an attempt to engage end users to participate in the implementation process. The enablers identified were the broad range of communication and promotional activities used to drive uptake, including attendance to routine clinical meetings and inductions, regular emails from various sources, and visual media placed around the hospital. Involving clinicians in testing and feedback sessions at an early stage of the change process also helped:

So on the whole that was good, the amount of feedback that we got back. And it helped iterate the product.

Participant #14, Google Health

The barriers identified were the adoption challenge where the system was opt-in and not integral to any clinical workflows. This was partially due to the limited functionality of the app when first deployed and the slow iterative improvements and updates during the development cycle. Moreover, the app was solely available on iOS devices; therefore, the potential user base was restricted:

I think ultimately the user base is driven by the utility of the product. If the product is super useful and provides value, then people will use it. Any limitation in the number of downloads, in my mind, always reflects back to the core value offering of the product.

Participant #7, ICHNT

I think also, in many cases, I think a lot of clinical users can be quite tech averse. And maybe that's from previous experience through your existing systems they're currently having to use. So there can be that barrier around, this is just going to have to be another thing that I'm going to have to use and it's almost coming at it from a “you need to show me the value before I actively engage in helping use this, I don't want it to be another burden on my clinical time.”

Participant #12, Google Health

### Information Governance

Collaborating with industry partners as data processors raised information governance issues during the implementation process. Extra governance and precautions were required because of the public interest and scrutiny in the partnership, with proactive engagement with external bodies such as the ICO, National Data Guardian, and organizational legal teams:

We had to jump through more hoops, and we had to be a little bit more careful, because normally we'd have a process where we can sign off on systems and suppliers in a fairly straightforward manner. We tend to only engage with the ICO and National Data Guardian if we think there's a major problem.

Participant #8, ICHNT

Respondents commented that data protection and security aspects were handled well, especially during the migration of the data center to a cloud platform despite the significant delays it caused to the implementation process:

I think it's the rigour of the processes that we put in place around our information governance and I think we do have a very strong information governance capability within the organisation. I think it's been working collectively through that, but inevitably these things take time, don't they?

Participant #6, ICHNT

Comprehensive patient engagement was also identified as an enabler:

We need just to make sure that we were on board with the right messaging and we were engaging with all of the right partners, in addition to the public. We went out to the public in a number of different ways just to make sure we were transparent and in good faith, and really clear on the intent.

Participant #9, ICHNT

Difficulties in data extraction and assessing data quality from the EHR and the regulatory burden were noted to be barriers with the effect of delaying the implementation process. This involved extensive engagement over many months with a large number of clinical, technical, and legal stakeholders at the Trust to review data processing agreements and assess the quality, accuracy, and safety of the data being processed:

I think there was probably maybe slight frustrations on both sides [with regards to delays], but I think there was also recognition that we need to get this completely right, and it was much better to be delayed [...] than go fast and have another cycle.

Participant #2, Google Health

Furthermore, issues with BYOD policies and a change in plan midway through the development cycle to require an MDM solution for extra security were also noted to have delayed the implementation process:

I think had someone with a lot of experience in MDMs just been around, they could have just sorted it out in a week. [...] I think that's a lack of experience on both sides, probably.

Participant #7, Google Health

### Partnerships

Mutually reinforcing partnerships can help organizations with digital change. The working practices between the partners were frequently commented on by respondents. Enablers identified were the technical, implementation, user engagement, and clinical collaborative working groups that convened weekly and were established to oversee specific aspects of the project:

We've had a formal governance arrangement in place, which has built into it a series of meetings for different groups. We've got a Streams steering group that has met every six weeks. We've had a technical working group that has met fortnightly. We've had a programme management weekly meeting, and we've had very well-defined attendees and good regular attendance from the right people for those meetings. That has got us into quite a good cycle of good communication for particular areas.

Participant #5, ICHNT

Well-defined roles and responsibilities between partners were also established. The technical expertise of Google Health team members was used to develop and implement the app. Respondents felt that this was not something that the Trust would be capable of doing unilaterally:

I think most health systems and NHS organisations are, 'We should go out and partner with start-ups and established companies,' and I think that's the right approach.

Participant #2, Google Health

The established link and connections between the Trust and the University were used to engage academic experts in the implementation process. This enabled rigorous continuous evaluation and benefit realization of the app:

The university, particularly in this setting, were clinician scientists who have a feel for both the clinical practice and also the research.

Participant #11, ICL

Occasional siloed working practices among partners were identified as barriers, causing delays in the implementation and making the partnership model feel like a traditional supplier–client model rather than the development partnership that was envisaged:

If you want a development partner, you have to work in a much more integrated, collaborative manner, and they don't do that at the moment. They go away with an idea. They say they've done a lot of thinking, and this is what they're planning to do, [...] We need to be a development partner.

Participant #8, ICHNT

### Resourcing and Skills

The ability of an organization to support mobile transformation is dependent on the resources and skills involved in the project. The presence of a dedicated, full-time project manager to oversee day-to-day running of the deployment was a key enabler in this process:

I think what really helped was appointing a project manager at the Trust whose principal responsibility was to bring all the different competing teams at the Trust together, and act as a single point of contact and project manage it.

Participant #3, ICL

I really feel like the project management at Imperial is very good and it's often not the organisation or the project management that's lacking, it's just it takes a long time because there isn't enough resource to actually do some of the work that the project manager is organising.

Participant #7, Google Health

However, respondents noted that the project manager was the only dedicated resource at the Trust and, therefore, a major barrier was the lack of personnel, investment, expertise, training, and support to help with the implementation process:

I think if we'd have more resource we could have moved more quickly and we might have realised more of the original scope. I think it's been resource-constrained.

Participant #6, ICHNT

### Lessons Learned

#### Overview

**Figure 2:**
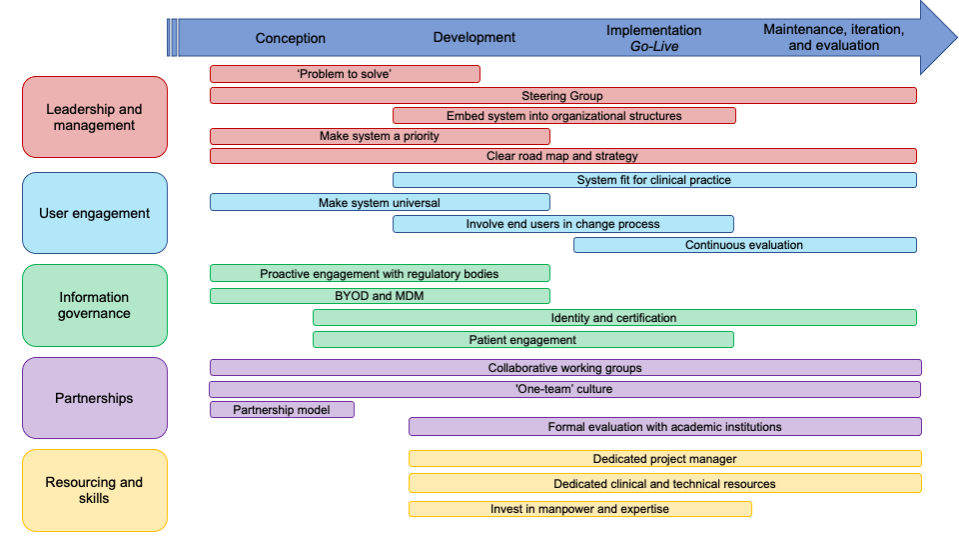
Proposed road map for large-scale, hospital-wide mobile implementation. BYOD: bring-your-own-device; MDM: mobile device management.

The implementation of mobile technology and working with commercial partners in NHS organizations were a significant challenge. The key lessons learned from this process are described in this section in addition to the proposed road map for the implementation of clinical mobile technology developed with commercial partners at scale. These are illustrated with temporal relationships in Figure 2*.*

#### Leadership and Management

##### Clarify the Problem to Solve

Be specific about the clinical problem that needs solving and assess at an early stage whether this problem can be ameliorated by mobile technology. This will help create a shared vision across the organization.

##### Make the System a Priority

Create a sense of urgency in the organization by making the deployment a priority among the leadership and end users.

##### Steering Committee With Senior Key Stakeholders and Clinical Involvement

Establish a board-level steering committee comprising high-level key stakeholders to guide the implementation. They should act as a decision-making body and build consensus on the strategic vision. This should include clinical leaders such as the CCIO and deputy CCIOs, which will help engage other clinicians and aid with dissemination.

##### Clear Road Map and Strategy

Implementation strategies need to be tailored to organizational circumstances and should be well-planned, allowing room for flexibility in timelines.

##### Embed Program Into Existing Organizational Structures

Ensure that mobile change is implemented as a key part of the organization’s digital transformation rather than as an isolated project and that it is interoperable with other systems already in place.

#### User Engagement

##### Choose a System That Is Fit for Clinical Practice and Focused on End Users

The system being deployed needs to be usable and effective for clinicians. It should fit into existing clinical workflows to improve the quality of care or efficiency. The value of the system should be demonstrated to clinicians to warrant adoption.

##### Make the System Universal

Choose a system that can be universally adopted. It should be device- and operating software–agnostic and offer functionality that will be useful to multiple clinical user groups.

##### Involve End Users in the Change Process

Give end users a sense of ownership over the change process and involve them in the iterative development of the product. A clinical user group should be established as a forum to discuss new features and contribute to user acceptance testing, and local champions should be enlisted. A variety of communication strategies to engage with end users to promote the system and ensure adoption should also be used. The strategy should be linked with wider Trust communications to help with distribution of promotional material.

##### Continuous Feedback to Evaluate and Inform Iterative Development

It is essential to capture user feedback on the system and respond in a timely manner. An agile, rapid turnaround should be targeted for iterative development to demonstrate to end users that their feedback is being regarded.

#### Information Governance

##### Proactive Engagement With Governance and Regulatory Organizations

If required, proactive engagement with the ICO and other regulatory bodies is recommended to ensure that all data processing, data security, and regulatory guidance are adhered to. Data processing, particularly if undertaken by industry partners, should be transparent and within legal boundaries.

##### Consider BYOD and MDM Policies at Early Stage

BYOD and MDM policies should be formulated to ensure security and privacy with widespread mobile implementation. If MDM is chosen, investment in the software will need to be considered. Tackling staff perceptions and attitudes toward MDM on personal devices will also need to be explored further before MDM is widely accepted.

##### Identity and Certification

The product should be embedded in the digital ecosystem of the NHS organization. The system should be made secure by using existing active directories for account creation and role-based access permissions.

##### Patient Engagement

Be open and transparent with patient and public groups about the nature of the partnership and data processing and security aspects.

#### Relationship Between Partners

##### Collaborative Working Groups With Defined Escalation Pathways

Clinical and technical work streams with defined roles and responsibilities should be established. These working groups should have defined terms of reference and clear escalation paths.

##### Frequent Communication Between Partners at All Levels

Frequent and effective communication channels between partners should be established to instill a *one-team* culture*.*

##### Decide on Partnership or Procurement Model

Appraise the nature of the partnership with the commercial supplier and decide whether a customizable system or an *off-the-shelf* system is required.

##### Partner With Academic Institutions to Perform Formal Evaluation

Pre-existing links with academic institutions should be used for continuous independent evaluation and benefit realization.

#### Resourcing and Skills

##### Dedicated Project Manager to Drive Through Vision and Act as Key Point of Call

Appoint a dedicated project manager who focuses on all activities related to clinical implementation. They should act as the key point of call to liaise with all stakeholders to help overcome any barriers.

##### Dedicated Clinical Implementation and Technical Teams

Ensure that resources are available for dedicated clinical implementation and technical teams within the NHS organization to support the implementation process.

##### Investment in Personnel and Expertise

Investments should be made in specialist expertise that may be required, personnel to aid implementation, training of staff, and ongoing support.

## Discussion

### Principal Findings

This study sought to (1) identify the enablers and barriers to the widespread implementation of mobile technology in an NHS Trust and (2) formulate an implementation road map from the experiences and perspectives of those leading and heavily involved with the change management process. In doing so, key enablers and barriers and the implementation road map were mapped onto 5 overarching themes that encompassed all the crucial aspects of the digital change management process: *leadership and management, user engagement, information governance, partnerships,* and *resources and skills* [16]*.*

We identified that, despite the implementation occurring in a Global Digital Exemplar NHS Trust with world-class IT infrastructure [23] in partnership with one of the world’s biggest technology companies and with strong support from an array of key stakeholders, the cultural and technological change required for mobile working and implementation in health care was a significant challenge. Widespread deployment of the mobile app was pursued at our organization; however, multiple barriers and hurdles were encountered along the process. These barriers were acknowledged to have either contributed to delays in the implementation or decreased the adoption of the app among end users. The key barriers identified were as follows: (1) delays to implementation (shifting prioritization, risk aversion, instigation of MDM policies and investment in software, problems with data quality and data extraction from the EHR, limited resources at the Trust, and the migration of data storage to a cloud-based platform) and (2) decreased adoption (limited functionality of the app that did not integrate into clinical workflows and was not clinically useful for large proportions of the workforce, the tardiness of iterative development and responding to feedback, and the fact that the app was not device-agnostic).

By considering the experiences and perspectives of key stakeholders in overcoming the aforementioned barriers, together with the enablers that were recognized to be present within the partnership, we proposed a novel implementation road map for mobile technology deployment at scale. This road map highlights the key lessons learned, which may act as a blueprint for multi-stakeholder scoping processes in health care organizations considering mobile transformation. This may help avoid some of the commonly encountered pitfalls and improve the likelihood of successful implementation of mobile technology.

This study also identified and exposed some of the difficulties NHS organizations may encounter if working with industry partners for digital change. With regard to Streams at ICHNT, added transparency about the partnership was required because of the media scrutiny and public interest [24]. This high-profile partnership led to some risk aversion on behalf of the NHS organization; however, the implementation of the General Data Protection Regulations and proactive engagement with regulators provided a secure backdrop for data processing. Furthermore, the relationship between the Trust and the industry partner was recognized as a development partnership rather than a supplier–customer partnership [25]. With this type of partnership being relatively novel and all partners having different ways of working, it is perhaps inevitable that frustrations were noted about the levels of collaborative working and the alignment of goals between the partners.

Although mobile change shares much similarity with digital change, it comes with its own unique challenges [26,27]. In this study, although many of the barriers can also be applied to digital change, additional mobile-specific barriers were identified, such as the adoption challenge and privacy and security concerns related to HCPs using their own smartphones for clinical purposes. Long-held beliefs about the appropriateness of mobile phones in hospital settings may be a further barrier [28,29]. This is particularly relevant with the prospect of HCPs using their personal smartphones for clinical purposes on a BYOD basis. This strategy can blur the lines between professional and personal use of smartphones, potentially creating conflicts that could arise related to their use in the wards or when off-site [30]. Furthermore, BYOD raises governance, information security, and patient confidentiality issues [27], which must be addressed. MDM software can be a solution to these concerns because of its ability to enforce security policies and secure devices [31]; however, implications such as the loss of control and privacy felt by staff and the financial investment required by the organization with this system may not be universally acceptable. Other options, including *hardening* the security of the app through features such as 2-factor authentication and geolocation, could be considered if MDM is not appropriate [32]. Overcoming these barriers is important to ensure widespread acceptability of mobile devices in clinical settings and requires increased awareness among both HCPs and patients as to the benefits of these technologies.

Although the cultural barriers may take more time and resources to overcome, there does now appear to be extensive and widespread progress in overcoming the technical and information governance barriers to mobile change identified in our study. The introduction of the General Data Protection Regulations in Europe has defined the legal framework for data processing, and guidance from NHSX [30] and the Medicines and Healthcare Products Regulatory Agency [33] has defined the current regulatory framework. The new EU Medical Device Regulation, implemented in 2021, will redefine the regulatory framework with more stringent and specific protocols for various types of medical device software and mobile apps [34]. A mobile technology investment toolkit was also recently published by NHS Digital that provided practical tools and resources for IT leads to deploy mobile technology [35]. Furthermore, the difficulties encountered in our study with data extraction, sharing, and interoperability will be alleviated with increased use of Fast Healthcare Interoperability Resources (a new international standard for health care data formats and elements) [36].

With increasing digital technology being introduced into the health care space and the potential introduction of advanced data-driven technologies, it is inevitable that NHS organizations will need to continue to work with commercial and industry partners to develop and implement interventions. This is now accepted, and the NHS relies on numerous strategic partnerships to improve outcomes and deliver its ambitions in all fields [37]. These partnerships need to be transparent and comply with legal, regulatory, and ethical boundaries [38] to ensure that the partnership is acceptable to patients and that they can trust it. However, the approach of technology companies in the health sector can be challenging as successful methods that have been used in other industries may not be appropriate in the regulated and necessarily risk-averse health care space. Maximizing new partnerships with technology companies requires the development of innovative interventions, the agile deployment of solutions in clinical environments, and ongoing evaluation and iterative development to improve the product [39]. Although more traditional medical device companies are limited in their speed of introducing products to market because of the time needed for design, safety testing, manufacturing, and efficacy trials, technology companies may lack these restraints and lean toward rapid iteration and updates to evolve and improve products. This can create opposing views on the balance between careful evaluation and thorough evidence-based principles, and rapid technology development and fast product cycles that need to be addressed [40]. Tension with other partners such as academic institutions may also ensue as comprehensive evaluations of a novel intervention inevitably require time and added cost, which must be accounted for in the product road maps of commercial companies.

### Limitations

The study was limited to a single NHS hospital and the implementation of a single clinical mobile app. Although this inevitably influenced the perspectives of the interviewees and many of the findings were related to local contextual factors, we believe that the broad sample of key stakeholders interviewed and robust qualitative analysis identified issues that are generalizable to the implementation of other mobile technologies in hospital settings. The sample size and heterogeneity of the participants was unavoidable because of the limited number of key stakeholders and members of the SSG suitable for inclusion in the study. However, purposeful sampling was used to ensure that a representative cross-section of the SSG was included in the study. Furthermore, we accept that there will be a bias with members of the SSG reviewing their own role in the implementation process. Viewpoints of end users of the app were not explored to compare and provide a *top-to-bottom* view of the mobile change management process. Finally, differences across various health care settings, hospitals, and departments were also not explored in this study.

### Conclusions

The implementation of mobile technology in health care and working with commercial partners has been a significant and complex challenge for NHS Trusts. With the requirement of more industry partnerships for advanced digital technologies in the future, the findings of this study should influence how other organizations undertake similar mobile transformations and improve the probability of successful implementation and widespread adoption. By overcoming the cultural and technological barriers identified and observing the proposed road map, future deployments of mobile technology in health care settings could be facilitated and have a greater chance of success.

## Abbreviations

BYOD: bring-your-own-device
CCIO: Chief Clinical Information Officer
EHR: electronic health record
HCP: health care professional
ICHNT: Imperial College Healthcare National Health Service Trust
ICL: Imperial College London
ICO: Information Commissioner’s Office
IT: information technology
MDM: mobile device management
NHS: National Health Service
SSG: Streams Steering Group
